# Occurrence of Isopenicillin-N-Synthase Homologs in Bioluminescent Ctenophores and Implications for Coelenterazine Biosynthesis

**DOI:** 10.1371/journal.pone.0128742

**Published:** 2015-06-30

**Authors:** Warren R. Francis, Nathan C. Shaner, Lynne M. Christianson, Meghan L. Powers, Steven H. D. Haddock

**Affiliations:** 1 Monterey Bay Aquarium Research Institute, 7700 Sandholdt Rd, Moss Landing, CA 95039, United States of America; 2 Department of Ocean Sciences, University of California Santa Cruz, Santa Cruz, CA, United States of America; 3 The Scintillon Institute, 6404 Nancy Ridge Dr., San Diego, CA 92121, United States of America; University of New South Wales, AUSTRALIA

## Abstract

The biosynthesis of the luciferin coelenterazine has remained a mystery for decades. While not all organisms that use coelenterazine appear to make it themselves, it is thought that ctenophores are a likely producer. Here we analyze the transcriptome data of 24 species of ctenophores, two of which have published genomes. The natural precursors of coelenterazine have been shown to be the amino acids L-tyrosine and L-phenylalanine, with the most likely biosynthetic pathway involving cyclization and further modification of the tripeptide Phe-Tyr-Tyr (“FYY”). Therefore, we searched the ctenophore transcriptome data for genes with the short peptide “FYY” as part of their coding sequence. We recovered a group of candidate genes for coelenterazine biosynthesis in the luminous species which encode a set of highly conserved non-heme iron oxidases similar to isopenicillin-N-synthase. These genes were absent in the transcriptomes and genome of the two non-luminous species. Pairwise identities and substitution rates reveal an unusually high degree of identity even between the most unrelated species. Additionally, two related groups of non-heme iron oxidases were found across all ctenophores, including those which are non-luminous, arguing against the involvement of these two gene groups in luminescence. Important residues for iron-binding are conserved across all proteins in the three groups, suggesting this function is still present. Given the known functions of other members of this protein superfamily are involved in heterocycle formation, we consider these genes to be top candidates for laboratory characterization or gene knockouts in the investigation of coelenterazine biosynthesis.

## Introduction

Bioluminescence is the emission of light due to a chemical reaction occurring within an organism and is widespread in the marine environment [1]. At least two components are typically involved: the first is a small molecule known as the “luciferin”, which is oxidized to produce light. The second is an enzyme that catalyzes the oxidation, typically called a luciferase or photoprotein, depending on the mechanism of activation [2]. Many luciferases and photoproteins have been cloned and sequenced, and in all cases, the proteins are encoded in the genome of the luminous organism, with species-specific variations in the primary sequence. Despite the breadth of enzymes, there is only a small set of light-emitting luciferins. Luciferins are different between bacteria, fireflies, and jellyfish (cnidarians and ctenophores), but within those three major types the same molecule is used by all species.

Although many genes have been identified for luciferases, the genetic origins of luciferins remain undetermined except for luminous bacteria. A remarkable case is the luciferin coelenterazine which is the most widely occurring luciferin in marine bioluminescence [2], its use being reported in at least nine phyla [1]. The chemical structure was determined in parallel by two groups, one working on the sea pansy *Renilla* and the other working on the hydrozoan *Aequorea* [3, 4]. The structure is composed of an imidazopyrazinone, a nitrogen-bearing heterocycle, with three side groups that correspond to amino acid side chains. Remarkably, this structure was highly similar to the *Cypridina* luciferin [5] (sometimes called vargulin), a luciferin used by a number of crustaceans. Despite structural similarity, the two luciferins do not appear to be interchangeable in the enzymatic reactions [6, 7].

Although coelenterazine was first extracted from *Aequorea*, it was later shown that *A. victoria* gets the molecule from its diet [8]. In fact, part of the widespread utilization of this molecule can be explained by its presence in marine food chains [8, 9], but it is unknown which range of species can synthesize it. Because of this, it is difficult to identify a biosynthetic pathway. Some studies have found strong evidence of biosynthesis in copepods [10] and decapod shrimp [11]. Additionally, other animals have been proposed as candidates based on reports of bioluminescence at early developmental stages. For example, a few very old reports had discussed “phosphorescence” from early-stage embryos of the ctenophores *Mnemiopsis leidyi* and a *Beroe* species [12, 13]. Various other reports had noted bioluminescence in embryos or early developmental stages [7, 14], suggesting the possibility that ctenophores indeed produce their own coelenterazine.

**Fig 1:**
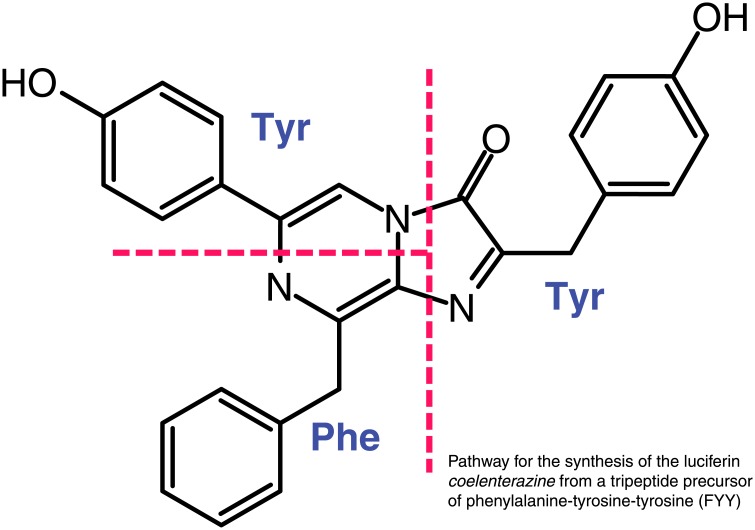
Structure of coelenterazine. Structure of coelenterazine showing the incorporation of the amino acids phenylalanine and tyrosine.

It had been proposed that the coelenterazine biosynthesis could involve three amino acids forming a tripeptide and then cyclizing [15]. Indeed, feeding experiments using stable isotopes have shown that in a copepod, coelenterazine was synthesized from phenylalanine and tyrosine [16], however the mechanism of this is unknown. Likewise, the structurally similar *Cypridina* luciferin is synthesized from arginine, isoleucine, and tryptophan [17]. These experiments only demonstrated the dependence on amino acids, which potentially could occur several ways. The most obvious mechanism would involve cyclization and further modification of the tripeptide Phe-Tyr-Tyr, the residues “FYY”, as a part of a larger peptide that is translated normally and subsequently cleaved and cyclized. Alternatively, it could be made by linking free amino acids, either to a series of enzymes which create di- and tri-peptide intermediates, then cyclize that into the final structure, or by a non-ribosomal peptide synthetase which links the residues and then cyclizes them in a fashion similar to the tripeptide that is converted into penicillin (Fig 1).

Here we searched for genes encoding “FYY” from the transcriptomes of luminous ctenophores. We were also interested in genes which could potentially perform the cyclization steps discussed above. We identified candidate genes that were present in the transcriptomes of luminous species and were not present for the non-luminous species. We compare these proteins to those from genomes of related animals and show that this group of proteins are highly conserved even among distantly related ctenophores, which is expected for critical biological processes.

## Results

### Sequencing and assembly of transcriptomes

We sequenced the transcriptomes of 21 luminous ctenophores and one non-luminous ctenophore (Table 1). Data from the genomes of two ctenophores, the luminous *Mnemiopsis leidyi* and the non-luminous *Pleurobrachia bachei* were used for comparison.

**Table 1:** List of ctenophores.

Species	Luminous? Y/N	Origin	Caught with	Extraction method	Library prep
*Bathocyroe fosteri*	Yes	Monterey Bay	ROV	QAP	TS-S-dT
*Bathyctena chuni*	Yes	Monterey Bay	ROV	QR	TS-dT
*Beroe abyssicola*	Yes	Monterey Bay	ROV	QAP	TS-S-dT
*Beroe forskalii*	Yes	Monterey Bay	ROV	QR	TS-S-dT
*Bolinopsis infundibulum*	Yes	Monterey Bay	ROV	QAP	TS-S-dT
*Charistephane fugiens*	Yes	Monterey Bay	ROV	QR	TS-S-dT
*Dryodora glandiformis*	Yes	Monterey Bay	Blue-water	QAP	TS-S-dT
*Euplokamis dunlapae*	Yes	Monterey Bay	ROV	QR	TS-S-dT
*Haeckelia rubra*	Yes	Monterey Bay	ROV	QAP	TS-S-dT
*Hormiphora californensis*	No	Gulf of California	Trawl	QR	TS-dT
*Lampea lactea*	Yes	Monterey Bay	Blue-water	Trizol	TS-dT
*Lampocteis cruentiventer*	Yes	Monterey Bay	ROV	QAP	TS-S-dT
*Ocyropsis maculata*	Yes	Gulf of California	Blue-water	QR	TS-S-dT
*Thalassocalyce inconstans*	Yes	Monterey Bay	ROV	QR	TS-S-dT
*Undescribed ctenophore B*	Yes	Monterey Bay	ROV	QR	TS-S-dT
*Undescribed ctenophore C*	Yes	Monterey Bay	ROV	QAP	TS-S-dT
*Undescribed ctenophore N1*	Yes	Monterey Bay	ROV	QAP	TS-S-dT
*Undescribed ctenophore N2*	Yes	Monterey Bay	ROV	QAP	TS-S-dT
*Undescribed ctenophore T*	Yes	Monterey Bay	ROV	QR	TS-dT
*Undescribed ctenophore V*	Yes	Monterey Bay	ROV	QR	TS-dT
*Undescribed ctenophore W*	Yes	Monterey Bay	ROV	QR	TS-S-dT
*Velamen parallelum*	Yes	Monterey Bay	Blue-water	QAP	TS-S-dT

Specimens and origins for ctenophores used in this study. See Methods for details on specimen collection. Abbreviations for extraction and library preps are: QAP, Qiagen AllPrep; QR, Qiagen RNeasy; TS-S-dT, TruSeq Stranded prep with oligo-dT selection; TS-dT, TruSeq with oligo-dT selection.

Transcriptomes were assembled for each organism using both Velvet/Oases [18, 19] and Trinity [20], the results were pooled and redundant sequences were removed (see Methods). In general, more sequences appeared to be full-length in the Trinity assemblies.

### Transcriptomes include a broad set of expressed genes

**Fig 2:**
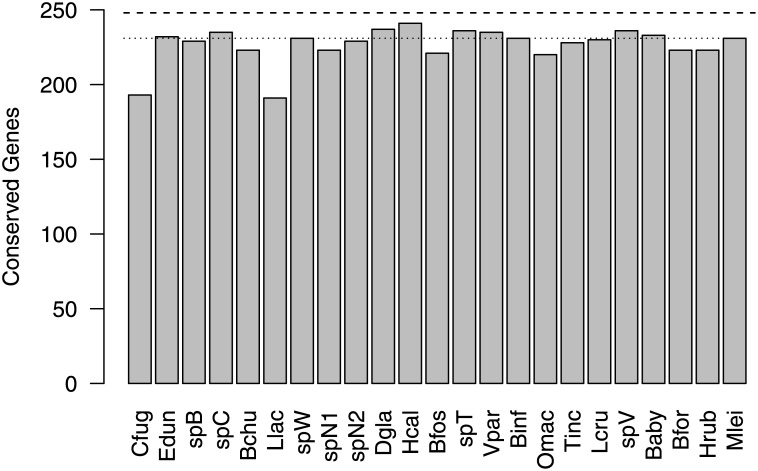
Survey of conserved genes across ctenophore transcriptomes. Dashed line indicates the maximum number of genes in this set, 248. The dotted line indicates the number of genes found in the *Mnemiopsis leidyi* genome. Most of the transcriptomes recovered a comparable number of genes as the genome. Species abbreviations are as follows: Bfos, *Bathocyroe fosteri*; Bchu, *Bathyctena chuni*; Baby, *Beroe abyssicola*; Bfor, *Beroe forskalii*; Binf, *Bolinopsis infundibulum*; Cfug, *Charistephane fugiens*; Dgla, *Dryodora glandiformis*; Edun, *Euplokamis dunlapae*; Hrub, *Haeckelia rubra*; Hcal, *Hormiphora californensis*; Llac, *Lampea lactea*; Lcru, *Lampocteis cruentiventer*; Mlei, *Mnemiopsis leidyi*; Omac, *Ocyropsis maculata*; Tinc, *Thalassocalyce inconstans*; spB, *Undescribed ctenophore B*; spC, *Undescribed ctenophore C*; spN1, *Undescribed ctenophore N1*; spN2, *Undescribed ctenophore N2*; spT, *Undescribed ctenophore T*; spV, *Undescribed ctenophore V*; Vpar, *Velamen parallelum*

Because the presence or absence of genes is difficult to address in transcriptomes, as they reflect only genes expressed at the time of extraction or freezing, we examined a large set of genes to support that the transcriptomes are complete. We have previously used a set of housekeeping genes to assess transcriptome completeness [21]. Compared to the numbers of full-length annotated genes found in the reference genomes, many of the transcriptomes appear to contain full-length homologs of over 80% of target genes (Fig 2). Thus, from the set of housekeeping genes, we extrapolated that the transcriptomes contained most essential genes and the presence or absence of genes may be due to factors of biology rather than sequence analysis.

### The FYY motif is found in the ctenophore genome

The ctenophore *Mnemiopsis leidyi* has been a model organism for bioluminescence for over a century. The genome was recently sequenced and is the first genome of a bioluminescent organism [22, 23]. We considered that one possible mechanism for coelenterazine biosynthesis may be from encoded “FYY” residues that are enzymatically cleaved. From the predicted 16,543 filtered gene models in the genome, we identified 374 gene products that contain the motif “FYY”. Two of these genes, ML199826a and ML35201a, had the FYY motif at the C-terminus of the protein. The two genes are highly similar (Table 2). The shorter of the two proteins, ML35201a, was 99% identical to the other (including gaps) varying only at a single residue but lacking a large piece of the N-terminus. Ignoring gaps, these two sequences were otherwise 100% identical (Table 2).

**Table 2:** Percent Identity Matrix of *Mnemiopsis* genes and proteins.

Gene	ML032920_35201	ML199826a	MLRB263543	MLRB263549	ML026010a	MLRB505111
ML032920_35201	=	97	93	94	54	51
ML199826a	100	=	91	94	52	50
MLRB263543	96	95	=	97	53	49
MLRB263549	97	97	98	=	56	50
ML026010a	48	46	45	47	=	49
MLRB505111	36	33	33	35	37	=

Pairwise identity for the *Mnemiopsis* genes. Protein sequence identity is shown on the lower portion and nucleotide sequences on the upper portion.

**Fig 3:**
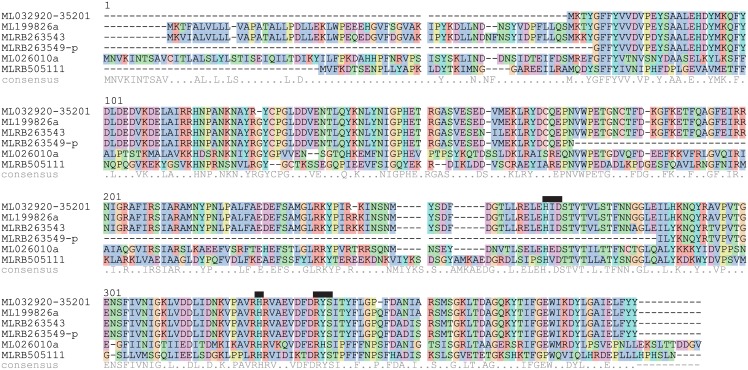
Multiple sequence alignment of ***Mnemiopsis*** proteins. ML032920-35201 is the putative full-length protein that connects ML032920a and ML35201a. MLRB263549-p indicates it is a partial sequence, as exons are missing in the scaffolds. The consensus sequence is indicated below, where identical residues are shown by ‘*’ and similar residues are shown by ‘.’. Black boxes indicate the highly conserved residues putatively involved in iron and 2-oxoglutarate binding.

We then examined the unfiltered gene models of *M. leidyi* and found two additional FYY-containing gene products in tandem on scaffold ML2635. The first one (MLRB263543) appeared to be complete and the second one (MLRB263549) was incomplete, as several exons were clearly missing. Based on the alignment to the other proteins (Fig 3), some of the missing exons would fall in regions with low sequencing coverage, represented only by “N”s in the genomic scaffold. The two proteins appeared to be nearly identical to each other, varying at three residues. Thus, we found two complete genes and two incomplete genes with the FYY ending.

### Four complete genes are annotated in *M. leidyi*

Because the predicted protein of ML35201a (the incomplete -FYY protein from the filtered models) does not start with methionine, and it is the first gene in its scaffold, we considered that the missing N-terminus may be due to incomplete annotation and searched for other pieces of the gene. The unfiltered protein models (MLRB35201) and Cufflinks assembly (ML3520_cuf_1) show an additional exon at the N-terminus. Since these genes still would be missing almost 100 amino acids compared to ML199826a, we then searched for the N-terminal fragment in other scaffolds, and recovered two unfiltered protein models (MLRB032948 and MLRB032949) and the corresponding filtered model fragment (ML032920a) at the 3′ end of scaffold ML0329. This suggests that scaffolds ML0329 and ML3520 are in proximity and are bridged by this gene. Using PCR, we were able to amplify a fragment of approximately 2kb using unique primers on each scaffold, confirming that these scaffolds are indeed adjacent (S1 Fig).

Examining possible cellular locations, SignalP [24] indicated that ML199826a is likely to be cleaved at the “ATA-LL” site of the N-terminus and possibly secreted (D score: 0.899), likewise for MLRB263543 (D score: 0.919). While the rest of the gene is nearly identical, the putative full gene (ML032920a-ML35201a) differs from ML199826a at the N-terminus. An identical piece to the N-terminus of ML199826a (residues “MKVIAL”) was found in ML0329, however if canonical splice sites are used, this would result in either a low similarity exon at the N-terminus or a stop codon, suggesting either that the genomic sequence is wrong, the gene is inactive due to a nonsense mutation, or that the N-terminal exons are unused for this gene. Given the very high identity scores for both the protein and gene, it is possible that the RNA support (Trinity and Cufflinks tracks) for the gene were actually due to mis-alignments of reads from ML199826a.

Another gene, ML026010a, was found to be similar to the FYY proteins (Fig 3 and Table 2) but lacked the FYY ending. Similarly, in the unfiltered models another homolog without the FYY was found (MLRB505111), which was different from both the FYY proteins and the other non-FYY protein (Table 2). This protein was not identified in the filtered models because it was split into two tandem pieces, ML50512a and ML50513a.

In all, there are four full-length annotated proteins and two incomplete proteins. As they are not entirely identical, they may be amenable to re-sequencing to verify the presence and expression of the incomplete genes.

### The FYY proteins are homologs of IPNS

To gain some insight as to the possible function of the FYY proteins, we compared the sequence to known proteins in various public databases. We BLASTed the FYY proteins against the nr (non-redundant) database on NCBI. Interestingly, nearly all of the top hits for all of the proteins were to a 2OG-Fe(II) oxygenase from the ciliate *Oxytricha trifallax* (Table 3). This was surprising since ciliates are unicellular eukaryotes and are not closely related to ctenophores. In a more restricted search using the Uniprot/Swissprot database, the top BLAST hits for many of the FYY proteins were to the same set of isopenicillin-N-synthase (IPNS) homologs, mostly from bacteria (Table 4). These proteins are members of a group of Fe-dependent oxygenases that include IPNS and deacetoxycephalosporin C synthase (DAOCS). These are the enzymes responsible for the heterocycle-forming steps of penicillin biosynthesis and the ring expansion in cephalosporin biosynthesis, respectively [25], and therefore were considered even stronger candidates for involvement in cyclization of FYY to coelenterazine.

**Table 3:** Top BLAST hits for FYY proteins in nr.

Hit	Species	Accession	ML032920-ML35201	ML199826a	MLRB263543	MLRB263549	ML026010a	MLRB505111
2OG-Fe(II) oxygenase	*Oxytricha trifallax*	EJY83212	2e-24	1e-24	2e-23	8e-5	6e-25	3e-16
2OG-Fe(II) oxygenase	*Oxytricha trifallax*	EJY68314	2e-17	2e-17	1e-17	–	2e-21	–
2OG-Fe(II) oxygenase	*Oxytricha trifallax*	EJY86133	1e-16	1e-16	3e-15	–	4e-27	8e-26
Isopenicillin N synthetase	*Crassostrea gigas*	EKC20116	5e-16	6e-16	1e-16	1e-5	3e-23	3e-23
Isopenicillin N synthetase	*Crassostrea gigas*	EKC29048	1e-15	1e-15	4e-15	–	2e-23	1e-21
Unnamed protein product	*Oikopleura dioica*	CBY23383	8e-15	1e-14	5e-16	–	2e-25	1e-19
Unnamed protein product	*Oikopleura dioica*	CBY34089	4e-14	3e-14	3e-15	–	3e-25	2e-19
2OG-Fe(II) oxygenase	*Oceanibaculum indicum* P24	ZP_11130131	2e-13	1e-13	–	7e-21	–	3e-14
Isopenicillin N synthase family	*Gordonia rubripertincta* NBRC 101908	ZP_11242214	1e-12	1e-12	–	–	–	–
2OG-Fe(II) oxygenase	*Mesorhizobium opportunistum* WSM2075	YP_004613268	2e-12	2e-12	–	–	1e-20	–
2OG-Fe(II) oxygenase family	*Campylobacter jejuni* 81116	YP_001482719	–	–	2e-12	–	–	–
2OG-Fe(II) oxygenase family	*Campylobacter jejuni* 414	ZP_06372273	–	–	2e-12	–	–	–
Putative iron/ascorbate-dependent oxidoreductase	*Campylobacter jejuni* ATCC 33560	ZP_14173854	–	–	5e-12	–	–	–
Putative isopenicillin N synthetase	*Talaromyces marneffei* ATCC 18224	XP_002152319	–	–	–	9e-4	–	–
Isopenicillin N synthase	*Mycobacterium phlei* RIVM601174	ZP_09977466	–	–	–	–	1e-20	–
2OG-Fe(II) oxygenase	*Mesorhizobium alhagi* CCNWXJ12-2	ZP_09292393	–	–	–	–	–	1e-14
Oxidoreductase	*Acidocella sp*. MX-AZ02	ZP_11251216	–	–	–	–	–	2e-14
Unnamed protein product	*Oikopleura dioica*	CBY11707	–	–	–	–	–	2e-13

Best ten BLASTP hits against the NCBI nr database for each of the proteins from *Mnemiopsis*. Numbers indicate e-values, for which a cutoff of 1e-3 was used. MLRB263549 was truncated and therefore did not align to many proteins.

**Table 4:** Top BLAST hits for FYY proteins in Swissprot.

Hit	Species	Accession	ML032920-ML35201	ML199826a	MLRB263543	MLRB263549	ML026010a	MLRB505111
Isopenicillin N synthase	*Streptomyces clavuligerus*	P10621	6e-12	8e-12	8e-12	–	2e-14	6e-12
Isopenicillin N synthase	*Lysobacter lactamgenus*	Q48739	2e-10	3e-10	1e-10	–	1e-17	4e-08
Isopenicillin N synthase	*Flavobacterium sp*. (strain SC 12,154)	P16020	1e-10	4e-10	1e-10	–	9e-18	2e-08
Isopenicillin N synthase	*Streptomyces griseus*	Q54243	4e-09	5e-09	1e-09	–	–	4e-07
Isopenicillin N synthase	*Streptomyces jumonjinensis*	P18286	5e-09	7e-09	4e-09	–	7e-15	1e-07
Isopenicillin N synthase	*Streptomyces microflavus*	P12438	1e-08	1e-08	2e-08	–	2e-11	–
Isopenicillin N synthase	*Streptomyces cattleya*	Q53932	1e-08	3e-08	2e-08	–	–	–
Isopenicillin N synthase	*Penicillium chrysogenum*	P08703	1e-05	1e-05	2e-06	–	1e-16	–
Isopenicillin N synthase	*Cephalosporium acremonium*	P05189	–	–	–	–	1e-17	–
Isopenicillin N synthase	*Emericella nidulans*	P05326	–	–	6e-05	–	7e-17	–
Isopenicillin N synthase	*Nocardia lactamdurans*	P27744	1e-05	2e-05	1e-05	–	8e-13	1e-11
1-aminocyclopropane-1-carboxylate oxidase	*Dictyostelium mucoroides*	A6BM06	–	–	–	–	1e-10	–
1-aminocyclopropane-1-carboxylate oxidase homolog 8	*Arabidopsis thaliana*	Q9M2C4	–	–	–	–	–	3e-07
Leucoanthocyanidin dioxygenase	*Petunia hybrida*	–	–	–	–	–	7.33e-07
1-aminocyclopropane-1-carboxylate oxidase homolog 10	*Arabidopsis thaliana*	Q9LSW6	–	–	–	–	–	9e-06
1-aminocyclopropane-1-carboxylate oxidase homolog 1	*Arabidopsis thaliana*	Q84MB3	–	–	–	–	–	9e-06
Gibberellin 2-beta-dioxygenase	*Arabidopsis thaliana*	Q9XFR9	7e-05	6e-05	–	–	–	–

Best BLASTP hits against the Uniprot/Swissprot database for the FYY proteins from *Mnemiopsis*. Numbers indicate e-values, for which a cutoff of 1e-3 was used.

Several conserved binding-pocket positions in the FYY proteins were detected when compared to the structures of IPNS and DAOCS [26, 27]. In ML199826a, we identified the iron-binding positions, H245, D247, and H301, suggesting that this function is still present (Fig 3). We also identified the conserved RXS motif at R310-S312, involved in coordinating the 2-oxoglutarate in DAOCS or the carboxyl group of valine in the tripeptide (ACV) in IPNS. Y221 was also a conserved residue that coordinates the ACV-valine in IPNS, however the same tyrosine in DAOCS points the opposite direction towards a backbone helix.

### FYY proteins are expressed only in luminous species

**Fig 4:**
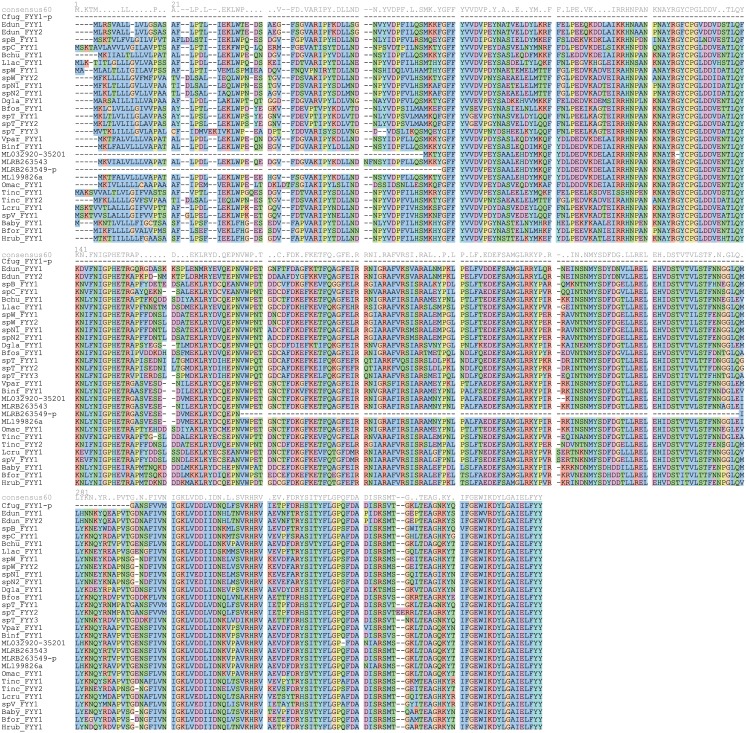
Multiple sequence alignment of all FYY proteins. Alignment of all FYY proteins across ctenophores. Partial sequences were excluded to show the high degree of identity, though they were used for subsequent analysis. The iron-binding residues are indicated by the black box above the consensus line. Species abbreviations are as follows: Bfos, *Bathocyroe fosteri*; Bchu, *Bathyctena chuni*; Baby, *Beroe abyssicola*; Bfor, *Beroe forskalii*; Binf, *Bolinopsis infundibulum*; Dgla, *Dryodora glandiformis*; Edun, *Euplokamis dunlapae*; Hrub, *Haeckelia rubra*; Llac, *Lampea lactea*; Lcru, *Lampocteis cruentiventer*; ML, *Mnemiopsis leidyi*; Omac, *Ocyropsis maculata*; Tinc, *Thalassocalyce inconstans*; spB, *Undescribed ctenophore B*; spC, *Undescribed ctenophore C*; spN1, *Undescribed ctenophore N1*; spN2, *Undescribed ctenophore N2*; spT, *Undescribed ctenophore T*; spV, *Undescribed ctenophore V*; Vpar, *Velamen parallelum*

We found a homolog of the FYY protein in nearly every ctenophore in our transcriptome set (Fig 4). In *Charistephane fugiens* we only found a partial sequence, though the assembly was among the worst of the set (Fig 2). Among the ctenophores examined here, only *Hormiphora californensis* and *Pleurobrachia bachei* have been reported to be non-luminous [28]. Because these ctenophores belong to a family of other non-luminous species (Pleurobrachiidae), we considered that this may be due to the genes being absent or unexpressed in that lineage. This was the only group within ctenophores that has been shown to be non-luminous and only contains a few members, so although it is a small sample they still make a fortuitous natural control against the large number of luminous species in this study.

**Fig 5:**
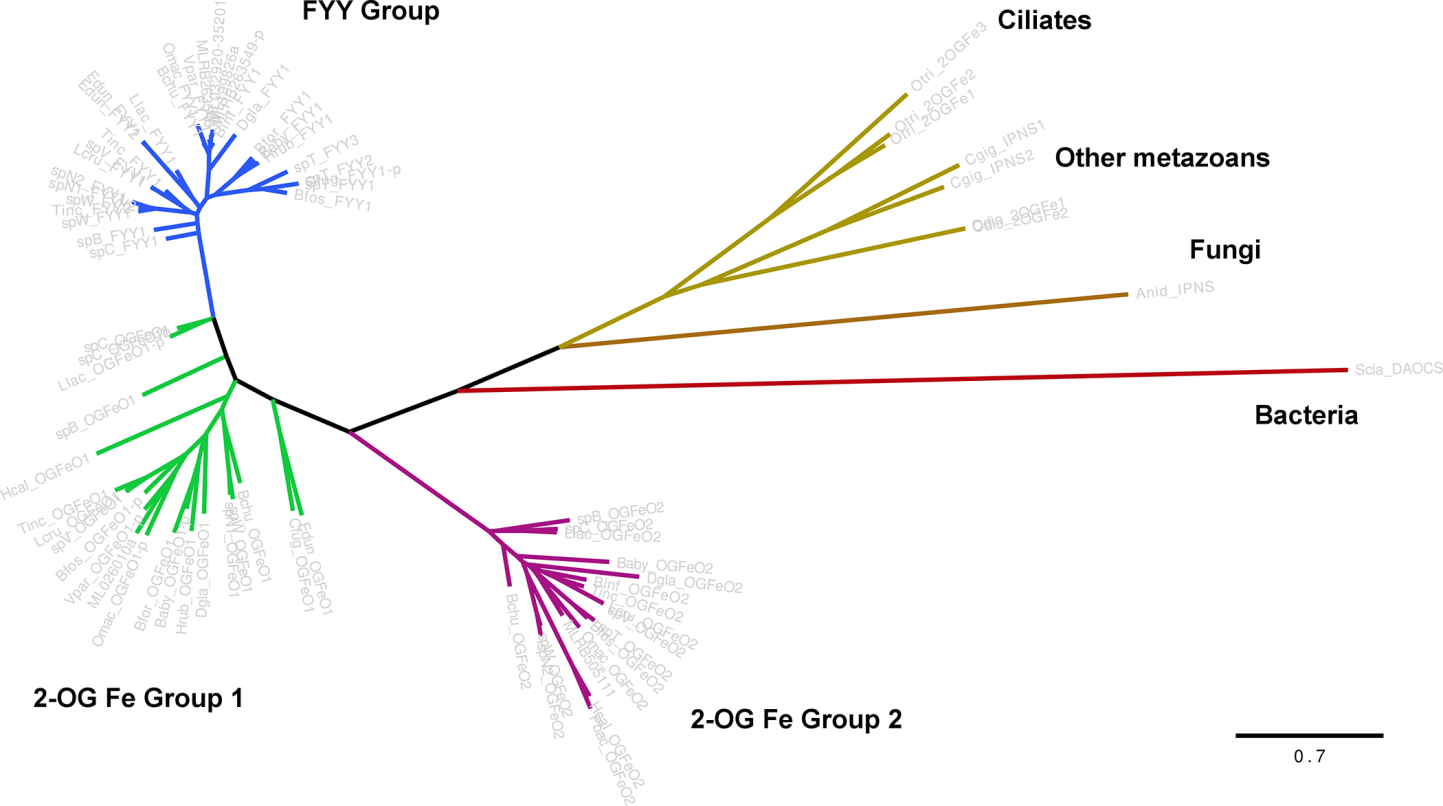
Maximum-likelihood tree of all putative ctenophore non-heme oxygenase protein sequences. Maximum-likelihood tree of all ctenophore non-heme oxygenase proteins including both FYY-containing (blue branches) and two non-FYY groups (green and purple branches). Outgroups from top BLAST hits (gold branches) and model enzymes (brown and red branches) show long branches compared to the FYY proteins. Sequence names are grayed out to emphasize branch lengths and clustering of the proteins. Scale bar indicates substitutions per site. Partial or incomplete sequences are indicated by -*p* as in Fig 4. Species abbreviations are as follows: Anid, *Aspergillus nidulans*; Bfos, *Bathocyroe fosteri*; Bchu, *Bathyctena chuni*; Baby, *Beroe abyssicola*; Bfor, *Beroe forskalii*; Binf, *Bolinopsis infundibulum*; Cfug, *Charistephane fugiens*; Cgig, *Crassostrea gigas*; Dgla, *Dryodora glandiformis*; Edun, *Euplokamis dunlapae*; Hrub, *Haeckelia rubra*; Hcal, *Hormiphora californensis*; Llac, *Lampea lactea*; Lcru, *Lampocteis cruentiventer*; ML, *Mnemiopsis leidyi*; Odio, *Oikopleura dioica*; Omac, *Ocyropsis maculata*; Otri, *Oxytricha trifallax*; Pbac, *Pleurobrachia bachei*; Scla, *Streptomyces clavuligerus*; Tinc, *Thalassocalyce inconstans*; spB, *Undescribed ctenophore B*; spC, *Undescribed ctenophore C*; spN1, *Undescribed ctenophore N1*; spN2, *Undescribed ctenophore N2*; spT, *Undescribed ctenophore T*; spV, *Undescribed ctenophore V*; Vpar, *Velamen parallelum*

Several BLAST searches (blastn, blastp, and tblastn) failed to identify a similar sequence to the FYY proteins in *Hormiphora* transcriptome, although the searches did find proteins similar to the non-FYY IPNS-homologs (S2 and S3 Figs). We considered that this absence could be due to a very low expression of the FYY protein which was removed during assembly. To address this, we then examined whether any fragments of the FYY proteins could be identified in the pre-assembled contigs (called “contigs.fa” by Velvet and “inchworm.K25.L25.DS.fa” by the first stage of Trinity.) We found 75 contigs this way and most were redundant when translated. Two putatively full-length proteins were identified from the contigs both of which group to non-FYY homologs in other ctenophores in the phylogenetic tree of the IPNS-homologs (Fig 5).

We then further examined the predicted genes from the *Pleurobrachia* genome [29]. As with *Hormiphora*, two different genes which are most similar to the non-FYY IPNS-homologs (sp2669069 to ML026010a and sp3466438 to MLRB505111) were found in the unfiltered models (Fig 5, S2 and S3 Figs). BLAST searches did not yield any sequence similar to the FYY proteins, nor were any of the conserved motifs found in any of the unfiltered models or translated adult mRNA datasets (RELEHXD, iron-binding site; GAIELFYY, conserved C-terminus). The absence of these proteins from our searches in the genome of *Pleurobrachia* and the transcriptome of *Hormiphora* indicated that these genes may have been lost in the *Pleurobrachiidae* clade. Without the genomic scaffolds to verify, we cannot resolve whether they were lost entirely or pseudogenized and unexpressed.

### Other luminescence genes are absent in *Hormiphora* and *Pleurobrachia*

**Fig 6:**
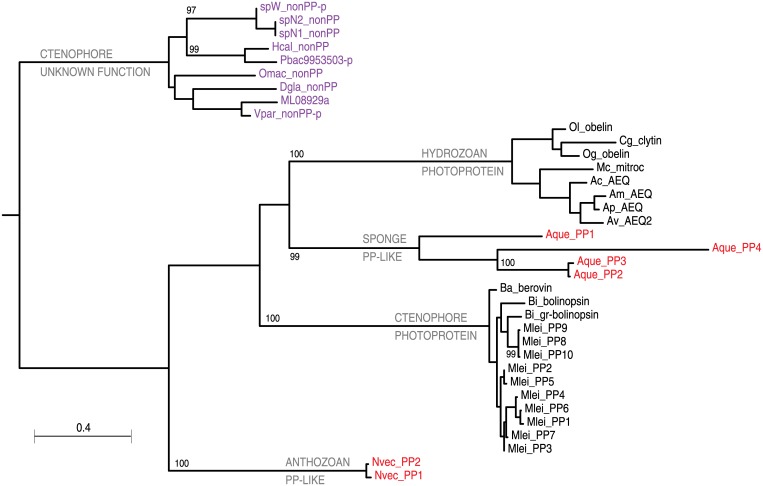
Maximum-likelihood tree of putative ctenophore photoprotein-like proteins. Maximum-likelihood tree of recovered ctenophore photoprotein-like genes and a set of verified cnidarian and ctenophore photoproteins from Schnitzler *et al*. (2012) [23]. Bootstrap values above 90 are shown. Abbreviations are as in Fig 5 with a few changes and additions: Ac, *Aequorea coerulescens*; Aque, *Amphimedon queenslandica*; Am, *Aequorea macrodactyla*; Ap, *Aequorea parva*; Av, *Aequorea victoria*; Ba, *Beroe abyssicola*; Bi, *Bolinopsis infundibulum*; Cg, *Clytia gregaria*; Mc, *Mitrocoma cellularia*; Nvec, *Nematostella vectensis*; Og, *Obelia geniculata*; Ol, *Obelia longissima*

While the lack of luminescence may be due to the absence of the FYY proteins, other proteins involved in the process may be responsible instead. One report suggests that even under several conditions, none of the members of the family *Pleurobrachiidae* including *Hormiphora* produced any light [28]. When tissue extracts from these species were incubated with coelenterazine, no light was detectable, suggesting that photoproteins are absent in these species [28]. Indeed, thorough searching in the transcriptome assemblies of *Hormiphora* only identified one putative photoprotein (Fig 6, S2 Alignment) which was closer in sequence to the non-luminous protein from *Nematostella vectensis* [23]. A homolog found in the *Mnemiopsis* genome is composed of four exons instead of one for all other photoproteins [23], suggesting it arose at a different time and may function in another way.

We then checked for photoproteins in *Pleurobrachia* and only found a partial gene of the homolog in *Hormiphora* (Fig 6) and no true photoproteins. Other hits to various photoprotein queries from other animals included two hits from Obelin (sb2644252, top hit back to hypothetical calmodulin-like protein; sb2643469, calmodulin), and one hit to a *Mnemiopsis* photoprotein (sb2667296, top hit back to NOX5, a calcium-dependent NADPH-oxidase), all due to the presence of EF-hand motifs.

We constructed a phylogenetic tree from these photoprotein-like genes in ctenophores and proper photoproteins from cnidarians and ctenophores, which show a clear difference between these photoprotein-like genes and true ctenophore photoproteins (Fig 6). True photoproteins are closer in sequence to cnidarian photoproteins than to these photoprotein-like genes, suggesting that duplication of the common ancestor of the two gene sets was before the divergence of metazoans. As the putative photoprotein-like genes in these three species lack the canonical EF-hand residues for calcium binding in photoproteins, it is questionable whether these proteins bind calcium at all. It is therefore likely that these putative genes are not photoproteins and perform some other function unrelated to bioluminescence. Ultimately, because we were unable to identify any photoproteins in the transcriptome of *Hormiphora* or the genome of *Pleurobrachia*, we conclude that those species are not bioluminescent in part because they lack photoproteins.

### The FYY proteins are highly conserved

Because long segments of the FYY proteins appeared to be identical across many ctenophores, we then measured the degree of identity and base substitution across the proteins. FYY proteins had much higher pairwise percent identities (Table 5) than either of the groups of the non-FYY proteins (Tables 6 and 7). The lowest amino-acid identity among the most distantly related members in the FYY group was 60% (average:71.61%) compared to 44% (average:56.00%) and 50% (average:62.17%) for non-FYY groups 1 and 2, respectively.

**Table 5:** Percent Identity Matrix of all FYY proteins.

Edun_FYY1	=	–	–	–	–	–	–	–	–	–	–	–	–	–	–	–	–	–	–	–	–	–	–	–	–	–	–	–
Edun_FYY2	95	=	–	–	–	–	–	–	–	–	–	–	–	–	–	–	–	–	–	–	–	–	–	–	–	–	–	–
spB_FYY1	66	66	=	–	–	–	–	–	–	–	–	–	–	–	–	–	–	–	–	–	–	–	–	–	–	–	–	–
spC_FYY1	67	66	75	=	–	–	–	–	–	–	–	–	–	–	–	–	–	–	–	–	–	–	–	–	–	–	–	–
Bchu_FYY1	65	65	71	70	=	–	–	–	–	–	–	–	–	–	–	–	–	–	–	–	–	–	–	–	–	–	–	–
Llac_FYY1	71	71	74	74	71	=	–	–	–	–	–	–	–	–	–	–	–	–	–	–	–	–	–	–	–	–	–	–
spW_FYY1	63	62	73	72	68	71	=	–	–	–	–	–	–	–	–	–	–	–	–	–	–	–	–	–	–	–	–	–
spW_FYY2	65	64	75	72	69	73	89	=	–	–	–	–	–	–	–	–	–	–	–	–	–	–	–	–	–	–	–	–
spN1_FYY1	65	65	74	70	69	74	85	91	=	–	–	–	–	–	–	–	–	–	–	–	–	–	–	–	–	–	–	–
spN2_FYY1	66	65	74	70	69	74	85	91	98	=	–	–	–	–	–	–	–	–	–	–	–	–	–	–	–	–	–	–
Dgla_FYY1	65	65	68	69	78	71	66	69	69	69	=	–	–	–	–	–	–	–	–	–	–	–	–	–	–	–	–	–
Bfos_FYY1	65	65	67	69	71	70	69	69	70	69	69	=	–	–	–	–	–	–	–	–	–	–	–	–	–	–	–	–
spT_FYY1	60	62	65	68	67	68	65	66	67	67	69	79	=	–	–	–	–	–	–	–	–	–	–	–	–	–	–	–
spT_FYY2	61	62	64	68	67	69	66	66	67	67	68	79	99	=	–	–	–	–	–	–	–	–	–	–	–	–	–	–
spT_FYY3	62	62	64	67	67	68	65	67	66	66	67	75	75	73	=	–	–	–	–	–	–	–	–	–	–	–	–	–
Vpar_FYY1	69	67	72	73	90	72	68	70	70	70	78	70	68	68	66	=	–	–	–	–	–	–	–	–	–	–	–	–
Binf_FYY1	66	64	70	72	91	71	68	70	69	69	79	70	67	67	66	94	=	–	–	–	–	–	–	–	–	–	–	–
ML032920_35201	71	69	73	77	89	73	72	73	71	71	82	73	70	70	71	93	93	=	–	–	–	–	–	–	–	–	–	–
MLRB263543	68	66	71	72	90	72	67	69	68	68	78	70	68	68	67	95	92	96	=	–	–	–	–	–	–	–	–	–
ML199826a	69	67	70	72	87	71	67	70	68	68	78	70	67	67	66	93	92	100	95	=	–	–	–	–	–	–	–	–
Omac_FYY1	64	63	68	70	88	67	66	66	65	64	74	68	66	66	66	84	84	89	85	83	=	–	–	–	–	–	–	–
Tinc_FYY1	68	68	74	74	71	75	73	76	74	73	70	69	66	66	68	72	73	75	72	71	69	=	–	–	–	–	–	–
Tinc_FYY2	65	64	75	72	70	75	86	96	90	90	69	71	67	68	68	70	71	73	70	70	67	76	=	–	–	–	–	–
Lcru_FYY1	66	66	74	73	70	74	70	72	73	72	68	71	70	70	68	71	70	71	70	70	68	80	73	=	–	–	–	–
spV_FYY1	66	66	74	73	68	73	71	72	73	72	67	70	70	70	66	71	69	71	70	69	68	79	72	95	=	–	–	–
Baby_FYY1	64	65	72	71	70	71	70	72	70	70	68	69	68	68	66	71	69	74	70	70	68	72	73	70	69	=	–	–
Bfor_FYY1	62	63	71	70	70	71	72	74	73	73	69	71	69	69	68	69	70	73	69	69	67	72	75	69	70	83	=	–
Hrub_FYY1	66	66	70	71	72	72	69	72	71	71	69	70	69	68	69	72	71	76	72	72	70	73	73	71	70	85	81	=

Pairwise percentage identity for the FYY proteins.

**Table 6:** Percent Identity Matrix of all 2-oxoglutarate Fe-Group 1 proteins.

Cfug_2OGFe1	=	–	–	–	–	–	–	–	–	–	–	–	–	–	–	–	–	–
Edun_2OGFe1	60	=	–	–	–	–	–	–	–	–	–	–	–	–	–	–	–	–
spC_2OGFe1	53	51	=	–	–	–	–	–	–	–	–	–	–	–	–	–	–	–
spC_2OGFe1b	53	51	100	=	–	–	–	–	–	–	–	–	–	–	–	–	–	–
spB_2OGFe1	54	52	58	57	=	–	–	–	–	–	–	–	–	–	–	–	–	–
Bchu_2OGFe1	51	54	57	57	56	=	–	–	–	–	–	–	–	–	–	–	–	–
spW_2OGFe1	52	53	56	56	56	64	=	–	–	–	–	–	–	–	–	–	–	–
spN1_2OGFe1	53	55	58	58	56	65	84	=	–	–	–	–	–	–	–	–	–	–
Hcal_2OGFe1	49	50	50	50	54	51	55	53	=	–	–	–	–	–	–	–	–	–
Pbac_2OGFe1	44	46	47	47	48	51	50	51	63	=	–	–	–	–	–	–	–	–
Dgla_2OGFe1	57	55	58	58	59	60	63	65	61	57	=	–	–	–	–	–	–	–
Omac_2OGFe1	49	47	51	51	48	57	55	57	51	47	67	=	–	–	–	–	–	–
ML026010a	48	48	52	52	52	57	58	58	53	47	65	59	=	–	–	–	–	–
spV_2OGFe1	48	51	49	48	48	59	60	61	51	50	66	61	58	=	–	–	–	–
Lcru_2OGFe1	51	52	52	51	53	61	61	62	53	53	66	63	61	95	=	–	–	–
Tinc_2OGFe1	47	49	49	48	50	58	60	60	52	49	67	58	61	79	80	=	–	–
Bfor_2OGFe1	48	50	54	54	52	56	60	61	52	49	65	58	59	59	60	58	=	–
Hrub_2OGFe1	50	52	54	54	51	60	58	59	52	49	64	56	56	57	58	58	64	=

Pairwise percentage identity for the 2OGFe1 proteins.

**Table 7:** Percent Identity Matrix of all 2-oxoglutarate Fe-Group 2 proteins.

spC_2OGFe2	=	–	–	–	–	–	–	–	–	–	–	–	–	–	–	–	–	–
spB_2OGFe2	63	=	–	–	–	–	–	–	–	–	–	–	–	–	–	–	–	–
Llac_2OGFe2	79	65	=	–	–	–	–	–	–	–	–	–	–	–	–	–	–	–
Bchu_2OGFe2	67	64	68	=	–	–	–	–	–	–	–	–	–	–	–	–	–	–
spW_2OGFe2	65	58	63	68	=	–	–	–	–	–	–	–	–	–	–	–	–	–
spN2_2OGFe2	65	58	63	67	86	=	–	–	–	–	–	–	–	–	–	–	–	–
Bfos_2OGFe2	64	59	63	65	68	68	=	–	–	–	–	–	–	–	–	–	–	–
Hcal_2OGFe2	56	51	56	57	58	56	55	=	–	–	–	–	–	–	–	–	–	–
Pbac_2OGFe2	57	50	58	55	57	57	56	75	=	–	–	–	–	–	–	–	–	–
Dgla_2OGFe2	58	54	55	57	61	61	61	55	54	=	–	–	–	–	–	–	–	–
spT_2OGFe2	64	59	63	66	68	68	84	55	57	59	=	–	–	–	–	–	–	–
Binf_2OGFe2	65	60	63	67	65	66	69	57	57	63	67	=	–	–	–	–	–	–
Omac_2OGFe2	64	57	60	64	64	63	66	55	57	60	65	71	=	–	–	–	–	–
MLRB505111	63	59	63	68	67	66	68	59	59	61	68	71	69	=	–	–	–	–
spV_2OGFe2	57	53	57	61	62	62	63	50	50	56	63	66	64	64	=	–	–	–
Lcru_2OGFe2	57	54	56	60	63	62	63	50	50	55	62	67	64	64	97	=	–	–
Tinc_2OGFe2	64	60	64	68	66	65	70	57	56	63	69	72	68	73	74	74	=	–
Baby_2OGFe2	61	58	60	61	61	61	62	53	54	56	63	59	61	63	58	58	64	=

Pairwise percentage identity for the 2OGFe2 proteins.

We then examined whether these genes were conserved across the ctenophore clade using codeml [30]. Due to the number of species with partial sequences, it was difficult to make clear statistical conclusions. Qualitatively, we found that FYY proteins were characterized by low ratios of non-synonymous to synonymous substitutions and generally much lower numbers of non-synonymous substitutions compared to the non-FYY proteins that were relatively more neutral (Table 8, S1 Table). Combined with the high identities across different ctenophore groups, this suggests that the FYY proteins are under strong purifying selection and any given mutation might result in the loss of activity for the protein, perhaps due to backbone changes which may affect a binding pocket or to interfaces with other proteins.

**Table 8:** Base substitution ratios for *Mnemiopsis* genes.

	ML199826a			MLRB263543			ML026010a			MLRB505111		
Species	dN/dS	dN	dS	dN/dS	dN	dS	dN/dS	dN	dS	dN/dS	dN	dS
spC	0	0	0.4271	0	0	0.9644	0.2061	0.3011	1.4612	0.7789	0.4126	0.5297
Hcal	0	0	0	0	0	0	0	0.2961	0	0.7218	0.5637	0.781
Pbac	0	0	0	0	0	0	0	0	0	0	0.5105	0
Dgla	0	0	0.4271	0	0	0.9644	0	0.2876	0	0	0.1506	0
Binf	0	0	0.2563	0	0	0.2563	0	0	0	0	0.1695	0
Vpar	0	0	1.6201	0	0	1.6201	0.3273	0.3244	0.9911	0	0	0
ML199826a	0	0	0	0	0	0.2577	0	0.5831	0	0.4804	1.092	2.2734
MLRB263543	0	0	0.2577	0	0	0	0.5407	0.5831	1.0785	0.8796	1.092	1.2415
Baby	0	0.0788	0	0	0.0788	0	0.1124	0.3499	3.1139	0.1821	0.182	0.9992
Bfor	0	0.1404	0	0	0.1404	0	0.0887	0.2071	2.3351	0	0	0
spV	0.0202	0.0792	3.9178	0	0.0792	0	0.0814	0.2063	2.5357	0	0.2292	0
Lcru	0.0202	0.0792	3.9178	0	0.0792	0	0	0.1839	0	0	0.2296	0
Edun	0.0235	0.038	1.6201	0.0235	0.038	1.6201	0.4249	0.4006	0.9429	0	0	0
spB	0.0393	0.0382	0.9738	0.0393	0.0382	0.9738	0	0.3139	0	0.3703	0.5359	1.447
spW	0.0408	0.0771	1.8886	0.0408	0.0771	1.8886	0	0.2075	0	0.446	0.4038	0.9053
Hrub	0.0464	0.0779	1.6773	0.0761	0.0779	1.0228	0	0.3183	0	0	0	0
Tinc	0.0552	0.0792	1.4356	0.0202	0.0792	3.9178	0	0.1158	0	0	0.1373	0
Bchu	0.0612	0.0385	0.6301	0.0268	0.0385	1.4356	0.3233	0.2546	0.7874	0	0.4472	0
Omac	0.0612	0.0385	0.6301	0.0268	0.0385	1.4356	0.3586	0.3413	0.9517	0	0.1401	0
spN2	0.0933	0.1394	1.4947	0.0933	0.1394	1.4947	0	0	0	0.2764	0.4	1.447
spT	0.1191	0.2111	1.7732	0.1191	0.2111	1.7732	0	0	0	0	0.0962	0
Llac	0.1224	0.0788	0.6437	0.0825	0.0788	0.9552	0	0.365	0	2.0579	1.3566	0.6592
Bfos	0.1406	0.1871	1.3311	0.0626	0.1871	2.9917	0.1943	0.1587	0.817	0	0.1798	0
spN1	0.1516	0.1394	0.9198	0.1516	0.1394	0.9198	0	0.2317	0	0	0	0

Base substitution rates of *Mnemiopsis* genes compared to those of other species. 0 indicates the model was inadequate for this analysis due to a lack of detected substitutions. Abbreviations are as in Fig 5.

## Discussion

Here we have sequenced and searched the transcriptomes of 22 ctenophore species for putative genes in the coelenterazine biosynthetic pathway. While it was previously demonstrated that coelenterazine can be synthesized from isotopically-labeled amino acids [16], several mechanisms could involve amino acids, including normal ribosomally-synthesized peptides. This led us to search for peptides including the motif “FYY”, and discovered proteins that were related to isopenicillin-N-synthases, a class of enzymes known for many heterocycle-forming reactions such as those which create the heterocyclic structure of the tripeptide penicillin. We have identified one family of genes across luminous ctenophores which both contain the residues “FYY” which occur in coelenterazine as well as having detectable similarity to non-heme iron oxidases. This includes several closely related genes in the genome of *Mnemiopsis leidyi* as well as two more distant non-heme oxidase families. These three protein families all appear to be closer to each other than to any other non-heme oxidases, which might be expected for an isolated clade such as the ctenophores.

This group of enzymes is poorly characterize in animals as their main observations were in bacteria and fungi for production of antibiotics. There was some precedent of a horizontal gene transfer event of a IPNS gene to an insect [31], however the results of the phylogenetic tree suggest that is unlikely in ctenophores (Fig 5). The evident conservation of the FYY proteins between species suggests that whatever the function is, it is very important to the physiology of the animals. Bioluminescence is known to have functional importance in ctenophores [32], and photoprotein genes appeared to be under tight purifying selection [23]. It could then be expected that the production of luciferin would be tightly controlled as well, as disruptions to either luciferin biosynthesis or photoproteins would result in a loss of bioluminescence.

Of the initial hypotheses of possible biosynthetic pathways, we were quite surprised to find two key characters in the same protein —that is, a FYY-containing protein that is also a non-heme iron oxidase. The apparent explanation is that, under some circumstance, these enzymes would be capable of auto-catalytic cleavage and cyclization of the C-terminal FYY residues to form coelenterazine. While there is no precedent for this type of reaction, it is evident from the types of chemistries displayed by other non-heme iron oxidases that the full range of activities of these enzymes is poorly characterized.

Verification of the functions could be realized two ways: cloning and knockout experiments. While cloning a gene is straightforward, expressing a functional protein is often challenging, given that the cofactors and conditions for activity are unknown. For example, because several slightly different isoforms were found in a few of the transcriptomes and the *Mnemiopsis* genome, it could be that multiple proteins are required for activity, perhaps as a hetero-dimer. These could, however, also just be redundant copies or very recent duplications in a species-specific fashion. Knockouts and other genetic manipulations would be ideal to confirm the overall involvement in a process, though one cannot easily discriminate functions without something like LCMS to confirm any intermediates. It was recently demonstrated that *Mnemiopsis* specimens could be maintained in the lab for generations [33], suggesting the possibility of genetic manipulations that may ultimately resolve the functions.

New genetically-encoded optical tools are always desired for potential cell biology applications. Coelenterazine, for example, is the substrate of the calcium-activated photoprotein Aequorin, yet its complex heterocyclic structure makes it expensive to produce synthetically and limits the use in reporter technologies. Because the biosynthetic pathways for all eukaryotic luciferins are still unknown or incomplete, both attempts to genetically engineer a eukaryote to be self-luminous have used codon-optimized versions of the bacterial Lux genes, one in tobacco plants [34], the other in cultured human cells [35]. Discovery of the biosynthetic pathway of coelenterazine would enable a broad range of novel reporter systems and may ultimately provide insights into the evolution of bioluminescence in marine systems.

## Materials and Methods

### Specimens and sequencing

Specimens were collected either by trawl net, during blue-water dives, or captured at depth using remotely-operated-underwater vehicles (ROVs) (Table 1). Invertebrate specimens were collected in the region bounded by 36° 44’ N 122° 02’W to the northeast and 35° 21’N 124° 00’W to the southwest. Operations were conducted under permit SC-4029 issued to SHD Haddock by the California Department of Fish and Wildlife. Species used are unprotected and unregulated, and no vertebrates or octopus were used, so the International and NIH ethics guidelines are not invoked, although organisms were treated humanely. All samples were frozen in liquid nitrogen immediately following collection. All specimens were sequenced at the University of Utah using the Illumina HiSeq2000 platform paired-end with 100 cycles.

### Transcriptome assembly

All computations were done on a computer with two quad-core processors and 96GB RAM. For each sample, raw RNAseq reads were processed as previously published [21]. Briefly, read order was randomized. Low-quality reads, adapters, and repeats were removed. For efficiency, subsets of reads were used to assemble transcriptomes. Assembly was done with both Velvet/Oases (v1.2.09/0.2.08) [18, 19] and Trinity (r2012-10-05) [20], though better sequences were often observed with Trinity. Transcripts from both assemblers were combined and redundant sequences were removed using the “sequniq” program in the GenomeTools package [36]. Ctenophore sequences used in analysis can be found at GenBank, with accessions: KM233765-KM233833. Raw transcriptomic reads for *Hormiphora californensis* are available at the NCBI Short Read Archive under accession SRR1992642.

### Genomic reference data

Gene models, scaffolds, and proteins for the *Mnemiopsis leidyi* genome [22] v2.2 were downloaded from NCBI at the *Mnemiopsis* Genome Portal (http://research.nhgri.nih.gov/mnemiopsis/). Gene models and transcripts for *Pleurobrachia bachei* genome v1.1 [29] were downloaded from the the Moroz Lab (http://moroz.hpc.ufl.edu/). Because the genomic scaffolds for *Pleurobrachia bachei* were unpublished, we did not analyze nucleotide sequences for this genome.

### Gene identification

All BLAST searches were done using the NCBI BLAST 2.2.28+ package [37]. Various *Mnemiopsis* genes were examined manually using the genome browser and in-house Python scripts (prealigner.py and fpaligner.py) which can be downloaded at the MBARI public repository (https://bitbucket.org/beroe/mbari-public/src).

### Alignments and phylogenetic tree generation

Alignments for proteins sequences were created using MAFFT v7.029b, with L-INS-i parameters for accurate alignments [38]. Trees for the IPNS-homologs and photoproteins were generate using RAxML-HPC-MPI v7.2.8 [39], using the PROTCATWAG model for proteins and 100 bootstrap replicates with the “rapid bootstrap” (-f a) algorithm.

### Purifying selection analyses

Pairwise percentage identity calculations were generated among a suite of output files using ClustalX. The program implements a simple calculation and ignores gapped positions. To assess for evidence of purifying selection, ratios of non-synonymous to synonymous substitutions (dN/dS) were calculated using codeml in the PAML v4.7 package [30]. The previously generated tree was used to provide branch topology. Other parameters were as follows: seqtype = 1 (codons); CodonFreq = 2 (the F3X4 model); model = 2.

### PCR amplification

PCR of ML032920a-ML35201a was performed as follows: 98°C for 1 min; 30 cycles of 98° for 10s, 56° for 15s, 72° for 60s; final extension phase of 72° for 7min. Reactions were 50*μ* L using Phusion High-Fidelity PCR Master Mix with HF Buffer (New England Biolabs). Primers used were: ML0329-end-F2 5′, CCA TGA AGA CTT ACG GAT TTT TCT ACG; ML3250-start-F 5′, GAG ATC AGG AGG AAC ATC GG; ML3250-R 3′, GGA GAA ACA GAA GAA AAA ACA TAC TGT TTA G. Genomic sequence failed to amplify when an alternate 5′ primer for ML0329-end-F1 (TTT CGT TAA TAG CTA TGA AGG TTA TCG C) suggesting there may be base errors. The 1% agarose gel containing 5*μ* L ethidium bromide was visualized and photographed under UV light. 5*μ* L of Quick-Load 1kb DNA Ladder (New England Biolabs) were used for band-size comparison.

## Supporting Information

S1 Fig Gel of PCR amplified genomic fragments from ***Mnemiopsis leidyi***. Amplification of gene ML35201a (right band) and the scaffold bridging ML032920-35201 (left band) with a 1kb ladder on the right.
(TIFF)

S2 Fig Multiple sequence alignment of all non-FYY group 1 proteins. Consensus line is shown above. Abbreviations are as in Figs 4 and 5.
(EPS)

S3 Fig Multiple sequence alignment of all non-FYY group 2 proteins. Consensus line is shown above. Abbreviations are as in Figs 4 and 5.
(EPS)

S1 Table Raw output from codeml. Unfiltered output of codeml to infer base substitution rates among all FYY and non-FYY proteins, as in Table 8.
(TXT)

S1 Alignment Clustal-format alignment of all ctenophore FYY proteins and outgroups. mafft-generated alignment of all ctenophore FYY and non-FYY proteins as well as outgroups, used to generate tree in Fig 5.
(ALN)

S2 Alignment Clustal-format alignment of all ctenophore photoproteins and outgroups. mafft-generated alignment of all ctenophore photoproteins as well as outgroups, used to generate tree in Fig 6.
(ALN)
