# Supplementary figures and images for: Occurrence of Isopenicillin-N-Synthase Homologs in Bioluminescent Ctenophores and Implications for Coelenterazine Biosynthesis
Source: PLoS One. 2015 Jun 30;10(6):e0128742. doi: 10.1371/journal.pone.0128742 (PMC4488382; doi:10.1371/journal.pone.0128742)

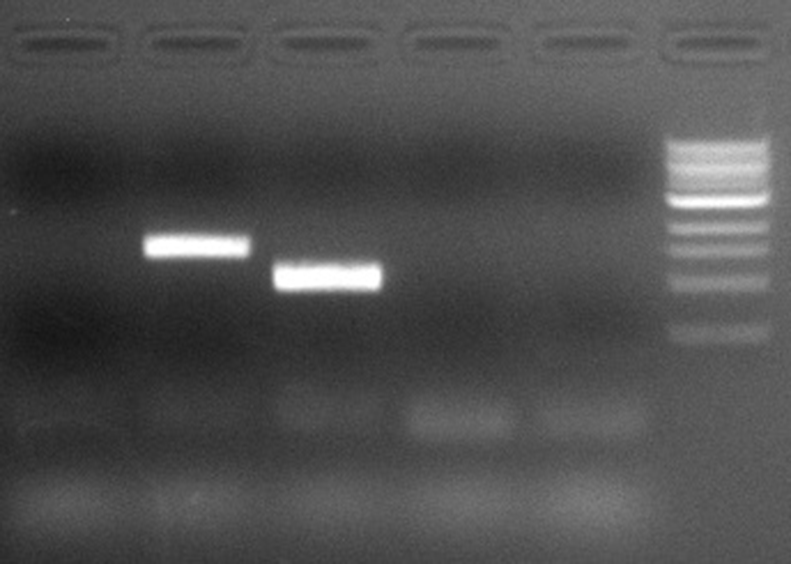

Supplement: S1 Fig — Amplification of gene ML35201a (right band) and the scaffold bridging ML032920-35201 (left band) with a 1kb ladder on the right. (TIFF) [file pone.0128742.s001.tiff]
